# Effects of snow cover on urban light climate environment in the high latitudes of northeast China

**DOI:** 10.1038/s41598-023-35825-x

**Published:** 2023-05-30

**Authors:** Fan Zhang, Nan Wang, Lijuan Zhang, Yue Chu, Shiwen Wang, Yutao Huang

**Affiliations:** 1 Key Laboratory of Land Surface Pattern and Simulation, Institute of Geographic Sciences and Natural Resources Research, CAS, Beijing, 100101 China; 2 Heilongjiang Province Key Laboratory of Geographical Environment Monitoring and Spatial Information Service in Cold Regions, Harbin Normal University, Harbin, 150025 Heilongjiang China

**Keywords:** Cryospheric science, Environmental impact

## Abstract

Light climate environment (LCE) has a significant impact on human health, behavioral characteristics, and the safety of life and property due to the high albedo of snow on the ground cover type, which in turn affects the regional climate and socio-economic development, but less relevant studies have been found. In this study, the effect of snow on daytime and nighttime light levels was quantified using comparative field observations and controlled experiments in artificial climate chambers, combined with analysis of variance and model fitting. The results of the study found that there was a significant difference between the presence and absence of snow on both daytime and nighttime light levels. During daytime, the ambient light level on the ground with snow is 5.68 times higher than without snow, an improvement of 12,711.06 Lux. At night, with moonlight, the nighttime illuminance with and without snow is 0.213 Lux and 0.01 Lux, respectively. When there is no moonlight, the snow has no significant effect on the light level. In addition, significant differences in LCE intensity with different snow depths, snow densities and black carbon (BC) pollution. At the same background light intensity, the LCE intensity varies significantly with increasing snow depth, snow density and BC pollution. The results reveals the quantitative impact of snow on LCE, providing scientific support for regional natural light energy use, human health and safety, urban environmental management and economic development.

## Introduction

Light climate environment (LCE) refers to natural conditions of outdoor natural light, which is a general term for the meteorology of natural light changes or variations^1^.Influenced by the solar motion trajectory, local light and climate characteristics, the LCE shows continuous variation over different spatial and temporal periods, which is reflected in the variation of illuminance values^2^. Snow is highly reflective to sunlight, and snow is more reflective than all types of the Earth’s surface cover^2^. Reflectivity of fresh snow and compact dry snow is as high as 86–95%, which is three to four times the surface albedo of grassland, and two to three times that of the forest cover^3–5^. In high mountain glacier areas, the high reflectivity of snow causes the retinas of humans who are not wearing sunglasses to be irritated by bright light, which can cause minor eye pain or temporary blindness, a phenomenon known medically as “snow blindness”^6,7^. Natural light is closely linked to human health^8–11^, with Rosenthal first suggesting in 1984 that a lack of natural light can cause a range of problems in the body, of which seasonal affective disorder (SAD) is a typical example^12^. Human biorhythms are influenced by light, which controls the secretion of melatonin by the pineal gland, which in turn affects sleep and mood^13,14^. Illuminance and spectral power distribution largely influence melatonin secretion^15^. If residential areas are exposed to such a strong among of reflective light environment for a long time, it can affect people's normal sleep and quality of life and also may cause various diseases, such as loss of vision, memory loss, and high incidence of cataracts^16,17^. Because of the presence of snow in midlatitude continental winters, the average reflectivity can exceed 60%^18,19^. Snow cover of high-latitude plays an important role in LCE because of its strong reflection^20–22^. Because of the existence of snow cover, quantitative research on the impacts of LCE have not been reported.

Recently, to save energy, consumers have begun to pay more attention to the utilization of natural light energy, and each major industrial country has invested a certain amount of research^23–25^. In the past three decades, many scholars have used mathematical methods to establish real-time luminous efficacy models to study the relationship between outdoor illuminance and solar radiation using actual measurement data^26,27^, including Lambert’s photometric theory^28^, illuminance distribution model of total cloud and clear sky^29^, average intermediate sky illuminance model, cloudy sky models, BRE sky illuminance model^30^, Tregnza’s random cloudy sky illuminance distribution model^31^, Perez’s full climate model^32^, Norio Igawa’s sky illuminance model^33^, and Kittler’s sky illuminance model^34–36^. Scholars have used the measured data from the Beijing LCE Observatory to build a corresponding LCE database based on illuminance data and to set up scalable data modules^37,38^. Additionally some scholars have discussed calculation methods, design methods, artistic treatments, and energy saving measures for natural and artificial light climates as well as light climate, light openings, light design, and light calculation methods^39,40^. Although scholars have conducted research from multiple perspectives on light climate calculations and design, snow has a significant impact on illumination due to its strong reflective effect on shortwave radiation^41,42^, but up to now, there is a lack of relevant quantitative research.

As the second largest stable area of snow in China, the snow season in the northeast can last up to six months^43^. Some studies have shown that the artificial light reflected from snow can be twice as bright as the full moon at its brightest^44,45^. Increased illumination values cause LCE changes that affect organisms, altering daily rhythms, migration patterns, and even reproductive cycles^46^. But because of strong disturbances from human activity, light-absorbing substances deposited on the snow surface darken the snow, resulting in reduced light levels^15,35,47^. Therefore, it is essential to investigate the impact of snow on the ambient LCE in northeast China. This study further enriched the research direction of ambient LCE by exploring the effect of snow on ambient LCE in northeast China through experiments. The results provide references to related research in geography, light environment, and interdisciplinary disciplines.

## Materials and methods

### Study area

**Figure 1:**
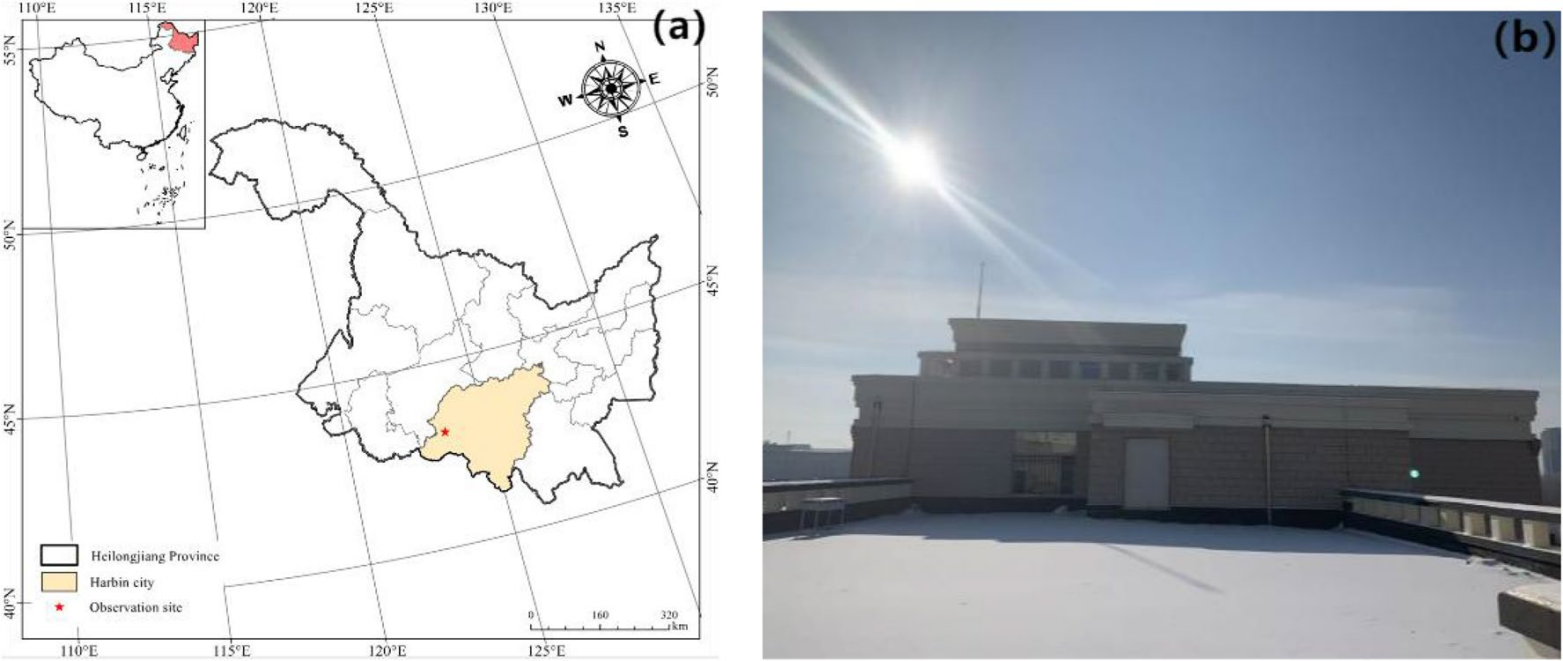
(**a**) Study area; and (**b**) the main observation site (In this figure (**a**) was created in the ArcGIS, version 10.8).

We selected observations to be carried out in Harbin, Heilongjiang Province, China. As the capital city of Heilongjiang Province, Harbin is located in southern Heilongjiang Province, 125°42′ E–130°10′ E and 44°04′ N–46°40′ N (Fig. 1a). With a permanent resident population of 988.5 million (up to 2021), it is the most populous city in northeast China with the largest spatial area. Harbin is a major city with high latitude and low temperature and has a midtemperate continental monsoon climate. Harbin is the capital city with the most abundant snow cover resources in China. Known as the “Ice City,” the average temperature throughout the year is 5.6 °C, the highest monthly average temperature is 23.6 °C, the lowest monthly average temperature is − 15.8 °C, the winter is long, and the summer is short.

The observation site for this study was established at Harbin Normal University, which is located in Songbei District, Harbin (Fig. 1b). The specific observation site is located on the east rooftop of the fifth floor of the Polytechnic No. 3 building. Artificial light does not influence this location, and it is influenced only by natural light, which enables observations of the outdoor ambient illuminance values under natural light conditions. In addition, the observation days in this study were chosen to be sunny, and the observation days were low cloud levels, and there were no effects such as haze.

### Data and methods

#### Observation methods

##### Observation of background illuminance values

We selected the three days of October 29, November 2, and November 5, 2020 as the lightness values of the snow-free ground. Observations were made at hourly intervals, positioned during the day from 6:00 to 18:00. To avoid the effects of refraction and scattering of the sun's rays, in this study, we identified the nighttime period from 22:00 to 4:00. Observations in the middle of the lunar month and the beginning of the lunar month were chosen in November to represent the background values with and without moonlight, respectively.

##### Daytime light-level observation with snow

**Figure 2:**
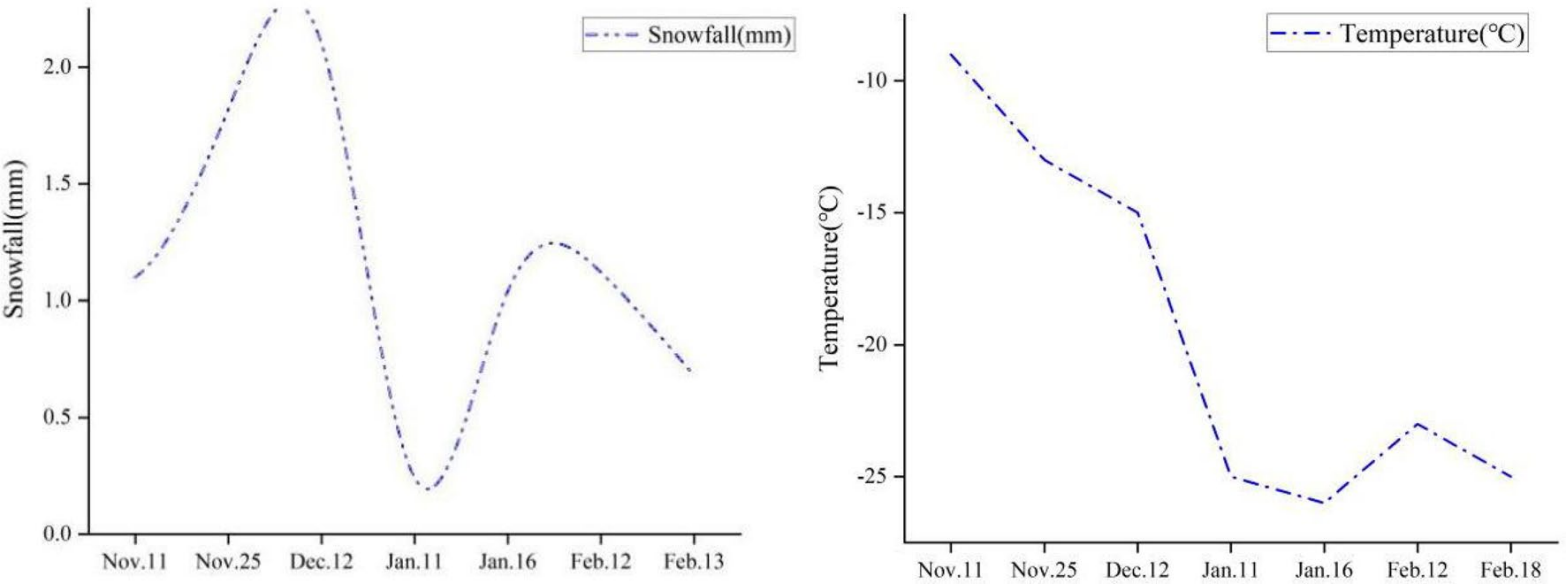
Snowfall and temperature distribution in Harbin during the observation period.

Measurements were taken at five-day intervals after the first snowfall on November 11 with an additional measurement taken in the event of a snow day. The period of observation was from November 11 (Lunar calender: October 7), 2021 to February 26 (Lunar calender: January 26), 2022, with a total of 22 days of measurements. Observations were made at hourly intervals for 24 h. During this observation period, there were seven snowfalls; the snowfall amounts and temperatures are shown in Fig. 2.

#### Observation equipment

**Figure 3:**
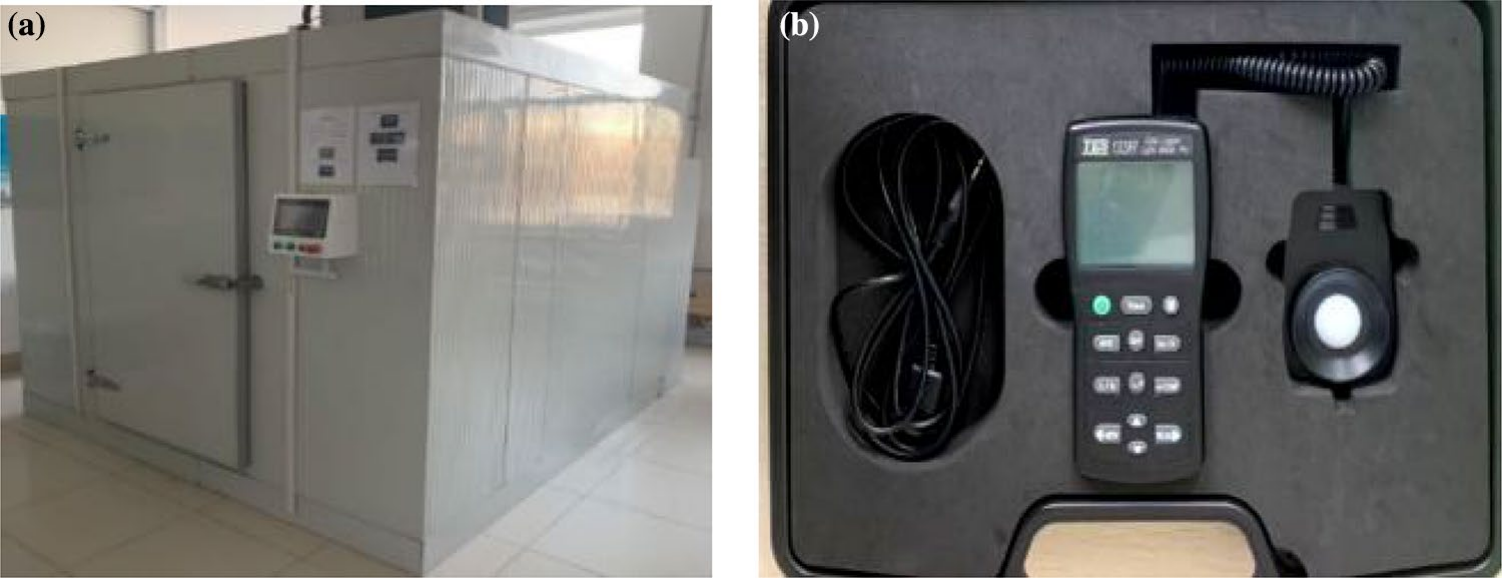
Observation equipment: (**a**) artificial climate box; (**b**) TES-1339R data recording professional-grade luminance meter.

In this study, the indoor tests mainly use artificial climate chambers to simulate the external climate environment by setting fixed environmental conditions. Climate chamber has Nanjing Jinheng experimental instrument factory production (RCO2-15M3-10D). The artificial climate chamber settings were adjusted before the start of each test, and the temperature was set to minus 25 °C. We used the TES-1339R Data Recording Professional-Grade Illuminance Meter (Fig. 3). TES-1339R is a high-precision digital illuminance meter often used in the field for illuminance measurements. Spectral reflectance standard specified by the International Illumination Association, this instrument has been patented by the United States. DES.446 The instrument has been granted a U.S. patent: DES.446, 135 and DES.469, 025, measurement range from 0.01 Lux to 999,900 Lux, which can automatically remove stray light and reflect the true illumination value of snow after direct sunlight. The illumination value reflected by the surface of the sun. Other test equipment included snow shovels, snow sieves, scales, common sieves, shovels, and rulers.

#### Snow sample collection

The test snow samples were collected on January 9, 2022 (first collection) and January 22, 2022 (second collection). The first snow sample was collected for the experiment on the effect of snow depth and density on ambient LCE conditions, and the second sample was collected for the experiment on the effect of snow density on ambient LCE conditions. The second snow sample was taken for the repeat test. The sampling ground is shown in Fig. 1a.

To minimize the effect of differences in direct sunlight and human disturbance on the basic properties of the snow, we collected snow samples from areas under both direct sunlight and the shade, as well as from areas where the snow surface was flat and untrampled. We ensured that the snow was uniform in nature. The snow was sieved into the test box using a snow sieve immediately after each sample was collected (thus ensuring that the test snow was uniform). This collection procedure ensured that the snow samples were of a similar density and that the test was carried out quickly.

#### Experimental design of indoor controlled conditions

The indoor tests were conducted using artificial climate chambers. We set up the chambers to simulate external climatic conditions by setting fixed environmental conditions. Before each test, we adjusted the settings of the artificial climate chamber to a temperature of − 25 °C.

##### Impact of snow depth on LCE

**Figure 4:**
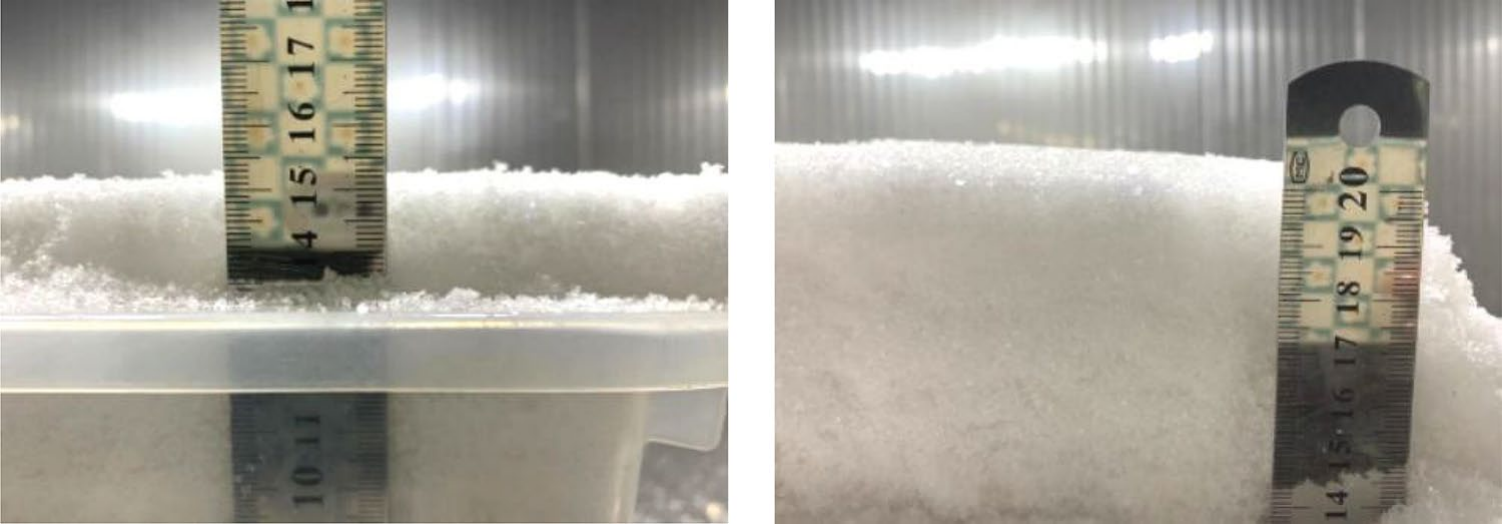
Experimental snow samples at different depths.

After setting the conditions of the artificial climate chamber, we adjusted the illumination level by changing the amount of light on the bare ground, and the initial illumination level in the environment was recorded for each of the five light levels as a background value for the ambient illumination. We designated four different snow depths of 5 cm, 10 cm, 15 cm, and 20 cm for the soil samples in the test chamber. The test was repeated three times (Fig. 4).

##### Impact of snow density on LCE

**Figure 5:**
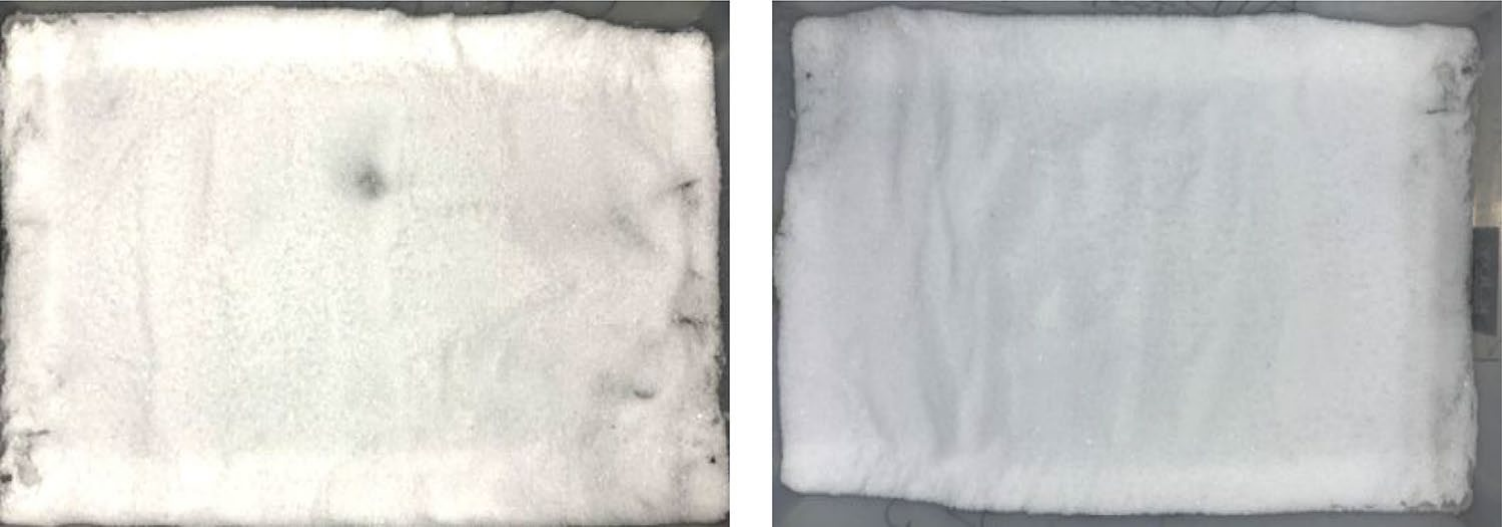
Experimental snow samples at different densities.

We set up indoor tests in an artificial climate chamber with separate settings of 0.01, 0.02, 0.03, 0.04, 0.05, 0.06, 0.07, 0.08, 0.09, and 0.10 g/cm^3^ of snow density, and adjusted the light illumination to record five light levels (100 W, 200 W, 300 W, 400 W and 500 W). We recorded the data three times for each set of observations and at different snow densities. Then the test was repeated three times (Fig. 5).

##### Impact of BC pollutants on LCE

**Figure 6:**
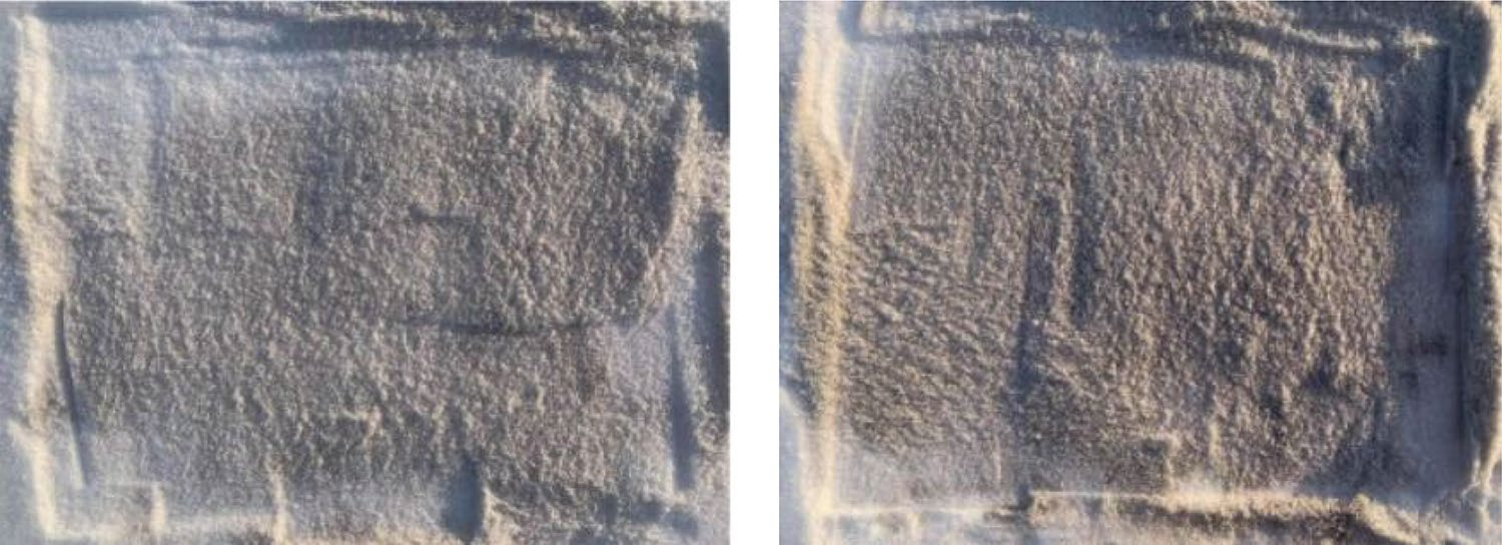
Experimental snow samples at different BC concentrations.

A total of three 1 × 1 m 2 test plots were set up on 22 January and 26 February 2022 at the outdoor observation site. Sprinkle 10 g, 20 g, 30 g, 40 g and 50 g of pollutant with snow and mix thoroughly. In the actual measurement data the central area is taken for the whole day and the ambient illuminance values are recorded for the different coverages (Fig. 6).

#### One-way analysis of variance

One-way analysis of variance (ANOVA) is used to investigate whether different levels of a control variable have a significant effect on an observed variable. In this paper, analysis of variance (ANOVA) is used in analyzing the differences in the effects of snow accumulation on ambient photoclimate for each parameter, mainly under different weather, different moon phases, each snow accumulation period, snow depth, and snow pollutant content on ambient photoclimate. With F test to determine whether the difference is significant. As follows:

1 $$SS_{A} = \sum\limits_{i = 1}^{{\text{r}}} {\sum\limits_{j = 1}^{k} {\left( {\overline{X}_{i} - \overline{X}_{ij} } \right)^{2} } }$$

2 $$SS_{E} = \sum\limits_{i = 1}^{{\text{r}}} {\sum\limits_{j = 1}^{k} {\left( {X_{ij} - \overline{X}_{i} } \right)^{2} } }$$

3 $$MS_{A} = \frac{1}{{\text{r - 1}}}\sum\limits_{i = 1}^{{\text{r}}} {\sum\limits_{j = 1}^{k} {\left( {\overline{X}_{i} - \overline{X}_{ij} } \right)^{2} } }$$

4 $$MS_{E} = \frac{1}{n - r}\sum\limits_{i = 1}^{{\text{r}}} {\sum\limits_{j = 1}^{k} {\left( {X_{ij} - \overline{X}_{i} } \right)^{2} } }$$

Each sample of random variables is called a group, and $\overline{X}_{i}$ is noted as the mean of group *i*. The mean of all $X_{ij}$ is called the total mean. The sum of the squared deviations of the mean of each group from the overall mean is the sum of the squared deviations between groups, reflecting the between-group differences, where $SS_{A}$ is the sum of the squared deviations of the mean of each group from the overall mean (i.e., the sum of the squared between-group deviations, reflecting the differences between groups; $SS_{E}$ is the sum of squared deviations within each group, reflecting the differences within each group; the degrees of freedom of $SS_{A}$ are $v_{1} = r - 1$; the degrees of freedom of $SS_{E}$ are $v_{2} = r\left( {{\text{k}} - 1} \right)$; $MS_{A}$ is the between-group mean squared difference; and $MS_{E}$ is the within-group mean squared difference.

Individuals with different levels of influence on LCE were independent of each other in this paper. Normality test, chi-square test and spherical symmetry test are required to test the data. A test level of 0.05 was used to test for normality at different levels of each influencing factor (Shapiro–Wilk Test). P-values greater than 0.05 at each level can be considered that each group of data is from a normal distribution overall.

#### Independent samples t-test

Independent samples *t*-test (no correlation existed between the experimental treatment groups; i.e., the independent samples), which is used to test the difference between the data obtained from two uncorrelated samples of subjects. In this study, we used a *t*-test to investigate and analyze the variability of light levels in the presence or absence of snow and in the presence or absence of moonlight during the day and night. The calculation is as follows:

5 $$t = \frac{{_{{\overline{{X_{1} }} - \overline{{X_{2} }} }} }}{{\sqrt {\frac{{(n_{1} - 1)S^{{_{1}^{2} }} + (n_{2} - 1)S_{2}^{2} }}{{n_{1} + n_{2} - 2}}\left( {\frac{1}{{n_{1} }} + \frac{1}{{n_{2} }}} \right)} }}$$

where $S_{1}^{2}$ and $S_{2}^{2}$ represent sample variances; and n_1_ and n_2_ are sample sizes.

## Results

### Effects of snow on daytime light levels

**Figure 7:**
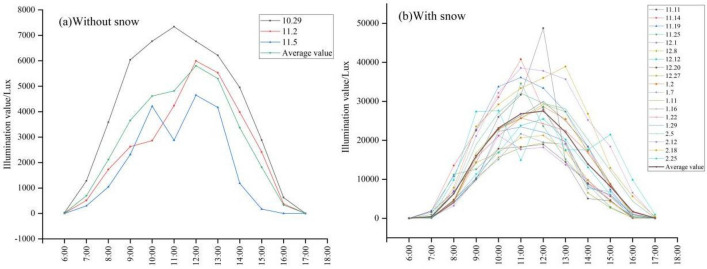
Characterization of hour-by-hour illuminance values in the environment (**a**) without snow, (**b**) with snow.

Three consecutive days of light level observations on a snow-free background (Fig. 7a). The results show a single-peaked trend during the day, with increasing ambient illuminance values from 6:00 to 12:00 and decreasing values from 12:00 to 17:00, with an average illuminance value of 2713.66 Lux and a maximum value of 5803.89 Lux at 12:00. The analysis of variance shows the three-day illuminance. According to the ANOVA, there was no significant difference between the three-day light levels, which indicated that the distribution of background illuminance values was stable. We made a total of 19 observations with snow (Fig. 7b). The results showed that the daytime variation was consistent with the distribution of illuminance without snow, but the average daytime illuminance value was 12,178.31 Lux, with a maximum value of 48,746.67 Lux, which was approximately 4.5 times greater than that without snow. These 19 observations were affected by cloud variations, which resulted in differences in the range of daytime illuminance distribution values.

**Figure 8:**
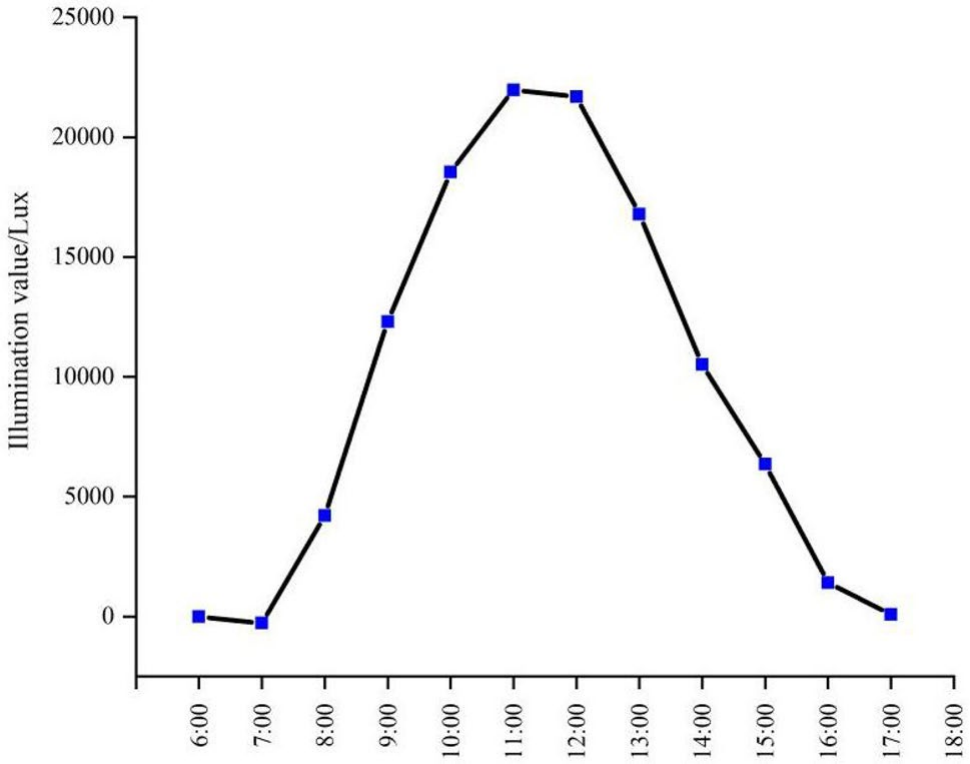
Difference diagram of snow environment illumination.

The values of the difference in ambient illumination with and without snow are compared (Fig. 8). The results show that snow has an average increase in light levels throughout the day of 4732.853 Lux and an average daytime increase of 9465.659 Lux. According to the *t*-test results, there was a significant difference (P < 0.01) between the effect of snow on ambient illuminance with and without snow, which indicated that snow had a significant effect on ambient illuminance. The effect of snow on ambient illuminance also exhibited daily variation, and the effect of snow on illuminance was inconsistent at different time points. This result indicated that the effect of snow on illuminance was related to the background illuminance.

As the solar altitude angle varies with local time and the declination of the sun, the solar altitude angle reaches its minimum value for the year on the winter solstice. In this study, the background illumination was taken in late October/early November, and the snow-lit observations lasted from November to February, with a large difference in solar altitude angle at the same time. Therefore, to analyze the effect of snow on light levels more precisely and to avoid errors caused by differences in ambient light levels influenced by differences in solar altitude angles, we made further comparisons under the same solar altitude angle background light level. Based on the actual situation in Harbin, the solar altitude angles were divided at five-degree intervals (Table 1).

**Table 1:** Ambient illuminance values with and without snow at the same range of solar altitude angles range.

Range of solar altitude angle	Without snow	With snow	Difference
− 5°–0°	14.070	113.173	99.103
0°–5°	698.511	3158.969	2460.458
5°–10°	2118.444	11,017.842	8899.398
10°–15°	3661.111	19,548.568	15,887.457
15°–20°	4616.666	25,639.474	21,022.807
20°–25°	4817.333	26,880.877	22,063.544
25°–30°	5803.889	27,668.246	21,864.357
20°–25°	5303.556	26,039.649	20,736.093
15°–20°	3372.444	23,008.947	19,636.503
10°–15°	1822.767	15,734.579	13,911.812
5°–10°	334.903	6149.930	5815.026
0°–5°	0.169	136.313	136.144
Average value	2713.655	15,424.714	12,711.059

**Figure 9:**
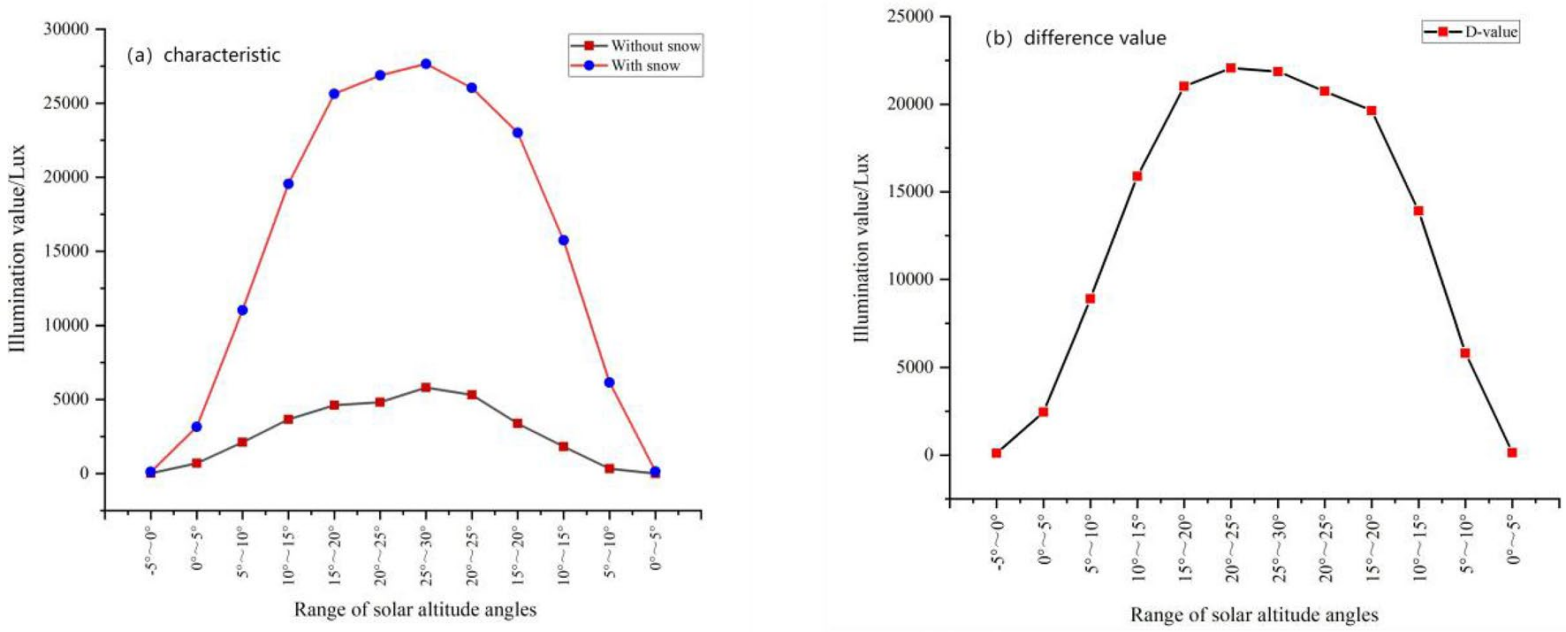
Ambient illuminance values with and without snow at the same range of solar altitude angles. (**a**) Characteristic; (**b**) difference value.

The average illuminance value during the period without snow was 2713.655 Lux and the average illuminance value during the period with snow was 15,424.714 Lux. Snow increased light levels by an average of 12,711.059 Lux, an increase of 468.41%, which was five times higher than that without snow, which indicated that snow had a significant impact on ambient light levels. The illuminance in the presence of snow was greater than the background value in all different solar altitude angle intervals, which increased with increasing solar altitude angle. The effect of snow on illumination varied with the intensity of the background light (Fig. 9). Strong snow reflecting light also was strong, background light was strong and weak, and snow reflecting light also was weak. Thus, we found further evidence of a significant enhancement of light levels by snow.

Further analysis showed that the effect of snow on illuminance essentially was distributed symmetrically with a quadratic parabolic curve as the solar altitude angle changes throughout the day:

6 $$Y = 393.005 + 5050.578X - 48.922X^{2} \;\;R^{2} = 0.836\;\;(P < 0.01),$$

From the equation we get:

$$d_{y} /d_{x} = - \;48.922.$$

This gives a rate of change in snow on lightness of 48.922 Lux/degree per unit increase in the solar altitude angle as the solar altitude angle increases in the presence of snow.

### Effects of snow on light levels at night

**Figure 10:**
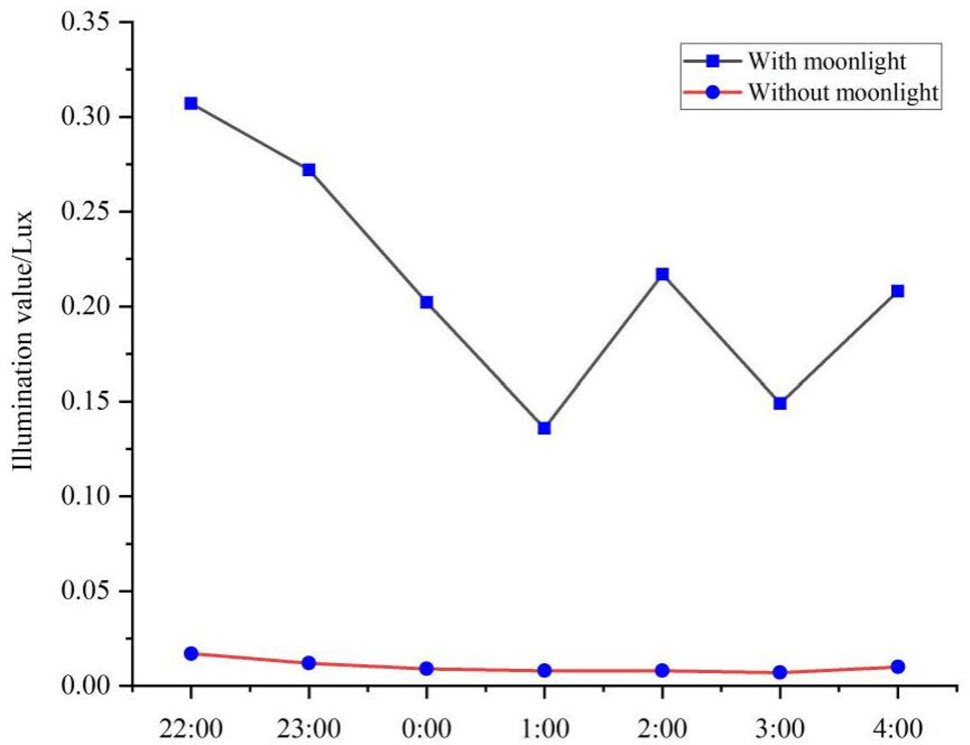
Characteristics of ambient illuminance values with and without moonlight when there is no snow.

In order to circumvent the effects of refraction and scattering of the sun's rays, this paper considers the period from 22:00 to the following day 4:00 is the night. Observations in the middle of the lunar month and the beginning of the lunar month were chosen in November to represent the background values with and without moonlight, respectively. Results for nighttime light-level background values are shown in Table 2 and Fig. 10. Nighttime illuminance was 0.010 Lux with moonlight and 0.005 Lux without moonlight (i.e., 0.005 Lux higher with moonlight than without moonlight). The distribution of illuminance from 22:00 to 4:00 had a significant difference on distribution. The *t*-test results showed a significant difference between illuminance with and without moonlight (P < 0.01).

**Table 2:** Ambient illuminance values with and without moonlight when there is no snow.

Time	With moonlight	Without moonlight	Average value
22:00	0.007	0.007	0.007
23:00	0.010	0.010	0.008
0:00	0.010	0.007	0.008
1:00	0.013	0.003	0.006
2:00	0.010	0.003	0.005
3:00	0.010	0.003	0.005
4:00	0.013	0.003	0.009
Average	0.010	0.005	0.007

**Figure 11:**
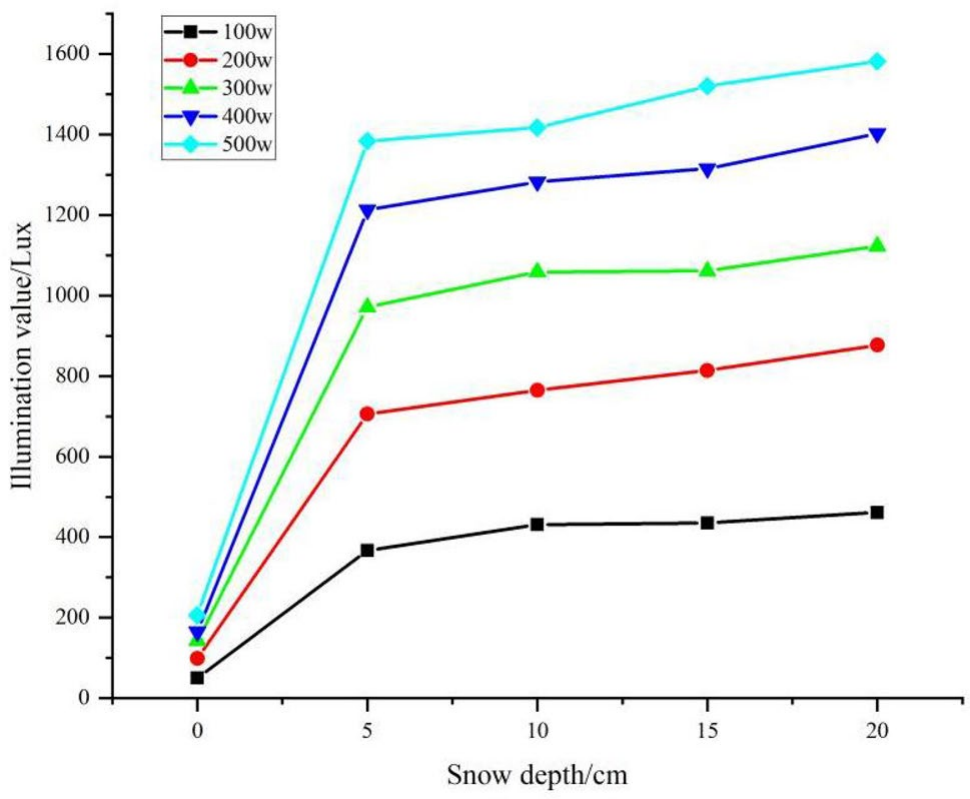
Characteristics of ambient illuminance values with and without moonlight when snow is present.

The distribution of light levels with and without moonlight when snow was present is shown in Table 3 and Fig. 11. Results showed that the nighttime illuminance with moonlight was 0.213 Lux and without moonlight was 0.010 Lux. When snow was present, the illuminance with moonlight was 0.203 Lux higher than without moonlight. We did not find a significant difference in the distribution of illuminance from 22:00 to 4:00. The results showed that the illuminance with moonlight when snow was present was significantly different from the illuminance without moonlight. We also observed a significant difference in illuminance (P < 0.01).

**Table 3:** Whether there is moonlight snow environment illumination value when there is snow.

Time	With moonlight	Without moonlight	Average
22:00	0.307	0.017	0.162
23:00	0.272	0.012	0.142
0:00	0.202	0.009	0.106
1:00	0.136	0.008	0.072
2:00	0.217	0.008	0.113
3:00	0.149	0.007	0.078
4:00	0.208	0.010	0.109
Average	0.213	0.010	0.112

According to this analysis, with moonlight, the nighttime illuminance with snow was greater than that without snow, the illuminance level ranges is 0.07–0.13 Lux, by an average of 0.203 Lux, Without moonlight, the nighttime illuminance with snow was greater than that without snow, by an average of 0.005 Lux. The difference with moonlight was greater than the difference without moonlight, and this difference was the same at each time point. The *t*-test showed a significant difference in illuminance with moonlight and with or without snow, as well as without moonlight and with or without snow (P < 0.05).

We compared the domain background values in the presence of snow (Table4) and concluded that snow increased nighttime illuminance by an average of 0.203 Lux in the presence of moonlight and 0.005 Lux in the absence of moonlight. The *t*-tests showed a significant difference in the increase in snow in the presence and absence of moonlight. This further indicated that snow had a significant effect on nighttime light levels. The distribution of light levels at each time point showed that there was no clear pattern. In the presence of snow, the increase in luminosity with moonlight was 20 times the background value, and without moonlight, the increase in luminosity was twice the background value.

**Table 4:** Difference in ambient illuminance values with and without moonlight and snow.

Time	With moonlight	Without moonlight
22:00	0.300	0.010
23:00	0.262	0.002
0:00	0.192	0.002
1:00	0.123	0.005
2:00	0.207	0.005
3:00	0.139	0.004
4:00	0.195	0.007
Average	0.203	0.005

### Effects of light-climate environment from snow cover factors

#### Effects of snow depth on the LCE

In this study, we investigated the effect of snow depth on illuminance through a two-factor interaction test (Table 5). The results showed that the illuminance with snow was greater than the illuminance without snow control—that is, the greater the snow depth, the stronger the ambient illuminance. Without snow, the average illuminance was 144.84 Lux, and when the snow depth was 5 cm, 10 cm, 15 cm, and 20 cm, the ambient light levels were 882.85 Lux, 969.2 Lux, 977.6 Lux, and 1073.35 Lux, respectively, which were 83.59%, 85.06%, 85.18%, and 96.51% higher than when there was no snow. It shows that snow depth is also is a factor in the illumination, indicating that the albedo varies with snow depth. The ANOVA results showed significant differences in snow depth, light level and snow depth*light level interactions.

**Table 5:** Analysis of variance (ANOVA) table for snow depth at different light levels.

Sources	Class 3 sum of squares	Freedom	Mean square	F	Significance
Modified model	17,851,347.422a	24	743,806.143	81,736.064	0.000
Intercept distance	52,171,220.378	1	52,171,220.378	5,733,039.820	0.000
Snow depth*Illumination	1,250,893.910	16	78,180.869	8591.212	0.000
Snow depth	9,425,664.141	4	2,356,416.035	258,944.047	0.000
Illumination	7,174,789.371	4	1,793,697.343	197,107.490	0.000
Error	455.005	50	9.100		
Total	70,023,022.805	75			
Total after amendment	17,851,802.427	74			

Further multiple comparisons of snow depths are shown in Table 6. The levels of 5 cm, 10 cm, 15 cm, and 20 cm were significantly different from each other at the 0.05 and 0.01 probability levels.

**Table 6:** Multiple comparison of different snow depths.

Dependentvariable: Illumination							
	Snow depth (I)	Snow depth (J)	Mean difference (I–J)	Standard error	Significance	95% confidence interval	
						Lower limit	Upper limit
LSD	0	10	− 896.39^a^	1.10	0.00	− 860.52	− 856.09
		15	− 858.30^a^	1.10	0.00	− 898.61	− 894.18
		20	− 956.25^a^	1.10	0.00	− 958.47	− 954.05
	10	5	− 795.31^a^	1.10	0.00	− 797.53	− 793.11
		0	896.39^a^	1.10	0.00	856.09	860.52
		15	38.08^a^	1.10	0.00	− 40.30	− 35.87
		20	− 59.86^a^	1.10	0.00	− 100.17	− 95.74
	15	5	101.07^a^	1.10	0.00	60.77	65.20
		0	858.30^a^	1.10	0.00	894.18	898.61
		10	− 38.08^a^	1.10	0.00	35.87	40.30
		20	− 97.95^a^	1.10	0.00	− 62.08	− 57.65
	20	5	62.98^a^	1.10	0.00	98.86	103.29
		0	956.25^a^	1.10	0.00	954.05	958.47
		10	59.86^a^	1.10	0.00	95.74	100.17
		15	97.95^a^	1.10	0.00	57.65	62.08
	5	5	160.94^a^	1.10	0.00	158.73	163.15
		0	795.31^a^	1.10	0.00	793.11	797.53
		10	− 101.07^a^	1.10	0.00	− 65.20	− 60.77
		15	− 62.98^a^	1.10	0.00	− 103.29	− 98.86
		20	− 160.94^a^	1.10	0.00	− 163.29	− 158.73

Based on measured average values.

^a^The significance level of the difference in means is 0.05.

**Figure 12:**
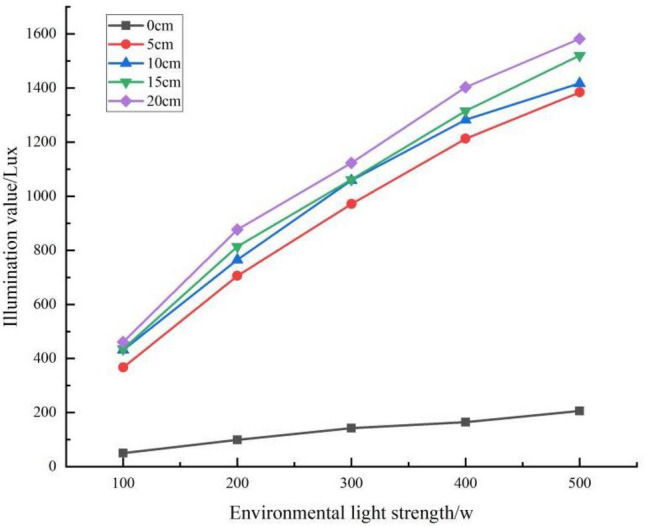
Comparison of illumination values for different snow depths under different light conditions.

The effect of different snow depths on light intensity illumination under different light conditions is shown in Fig. 12. The results showed that as the snow depth increased, the light intensity values followed an increasing trend.

A linear fit of snow depth to illuminance values was obtained, and the equation for the relationship between snow depth and illuminance values under different light conditions was best fitted as a linear function (Table 7). The results showed that the illuminance values increased rapidly at different light intensities as the snow depth increased from 0 to 5 cm, with the rate of increase decreasing significantly above 5 cm. Therefore, we compared the rates of change in segments. The rate of increase in illuminance from 0 to 5 cm was much greater than that from 5 to 20 cm. At 100 w, for example, an increase in illuminance of 63.346 Lux/cm was observed from 0 to 5 cm and 5.732 Lux/cm from 5 to 20 cm, which was 11.051 times greater than the latter. At 200 w, 300 w, 400 w, and 500 w, the rate of increase in illuminance was 13.270, 14.716, 17.386, and 16.929 times higher for 0–5 cm than for 5–20 cm, respectively.

**Table 7:** Equation for the relationship between snow depth difference and illumination value under different light conditions.

Snow depth/cm	Illumination/W	Equation	R^2^	Rate of change Lux/w
0–5	100	Y = 63.346x + 50.50	1.000^a^	63.346
	200	Y = 121.286x + 99.17	1.000^a^	121.286
	300	Y = 165.580x + 143.1	1.000^a^	165.580
	400	Y = 209.554x + 164.9	1.000^a^	209.554
	500	Y = 235.554x + 206.23	1.000^a^	235.554
5–20	100	Y = 5.732x + 352.23	0.924^a^	5.732
	200	Y = 9.140x + 649.72	0.999^a^	9.140
	300	Y = 11.252x + 939.165	0.946^a^	11.252
	400	Y = 12.053x + 1152.67	0.987^a^	12.053
	500	Y = 13.914x + 1301.83	0.984^a^	13.914

^a^The significance level of the difference in means is 0.01.

**Figure 13:**
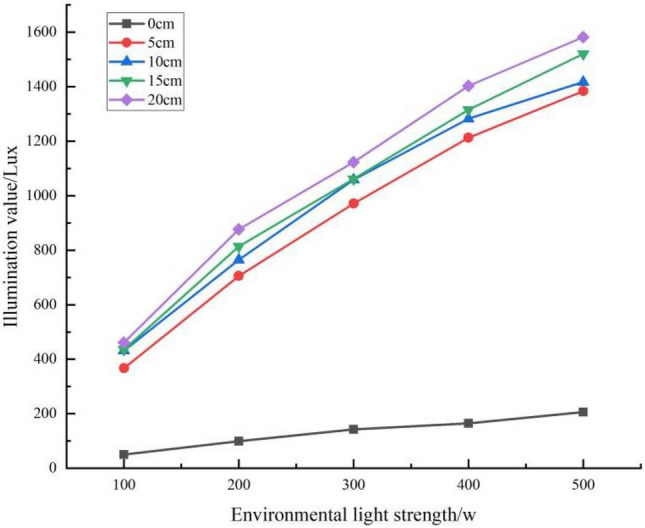
Effects of snow depth on illuminance values under different light conditions.

In addition, the light values for each snow depth at different light intensities are shown in Fig. 13. The results showed that the rate of increase with increasing light intensity also increased with snow depth for different snow depths. Further analysis shows that at the same snow depth, the rate of impact on ambient light levels is not consistent at different snow depths as the ambient light level increases. The greater the depth of the snowpack, the faster the rate of increase with increasing light intensity the faster the rate of increase (Table 8).

**Table 8:** Equation for the relationship between light and illumination values at different snow depths.

Snow depth/cm	Equation	R^2^	Rate of change Lux/w
0	Y = 37.719x + 19.623	0.993^a^	37.719
5	Y = 254.061x + 165.917	0.993^a^	254.061
10	Y = 248.952x + 244.232	0.987^a^	248.952
15	Y = 267.027x + 228.091	0.993^a^	267.027
20	Y = 276.644x + 259.110	0.990^a^	276.644

^a^The significance level of the difference in means is 0.01.

#### Effects of snow density on the LCE

By controlling the interaction test of snow density and light intensity, the results showed that under the same light intensity, the higher the snow density was, the stronger the ambient light intensity was. When the snow density was 0.01 g/cm^3^, the ambient light intensity was 910.48 Lux on average, and the ambient light intensity increased by 58.33%, when the snow density was 0.02, 0.03, 0.04, 0.05, 0.06, 0.07, 0.08, and 0.09 g/cm^3^, the ambient light level increased by 13.84%, 25.43%, 31.83%, 35.93%, 36.68%, 38.07%, 38.61%, and 39.42%, respectively. Analysis of variance showed significant differences between snow density and light levels in all cases (P < 0.05) (Table 9).

**Table 9:** Equation for the relationship between snow density and illumination values under different light levels.

Illumination/W	Equation	R^2^
100	Y = 74.071In(x) + 531.558	0.990^a^
200	Y = 138.775In(x) + 1030.683	0.994^a^
300	Y = 194.049In(x) + 1453.117	0.987^a^
400	Y = 203.380In(x) + 1637.197	0.993^a^
500	Y = 228.792In(x) + 1873.009	0.990^a^

^a^The significance level of the difference in means is 0.01.

**Figure 14:**
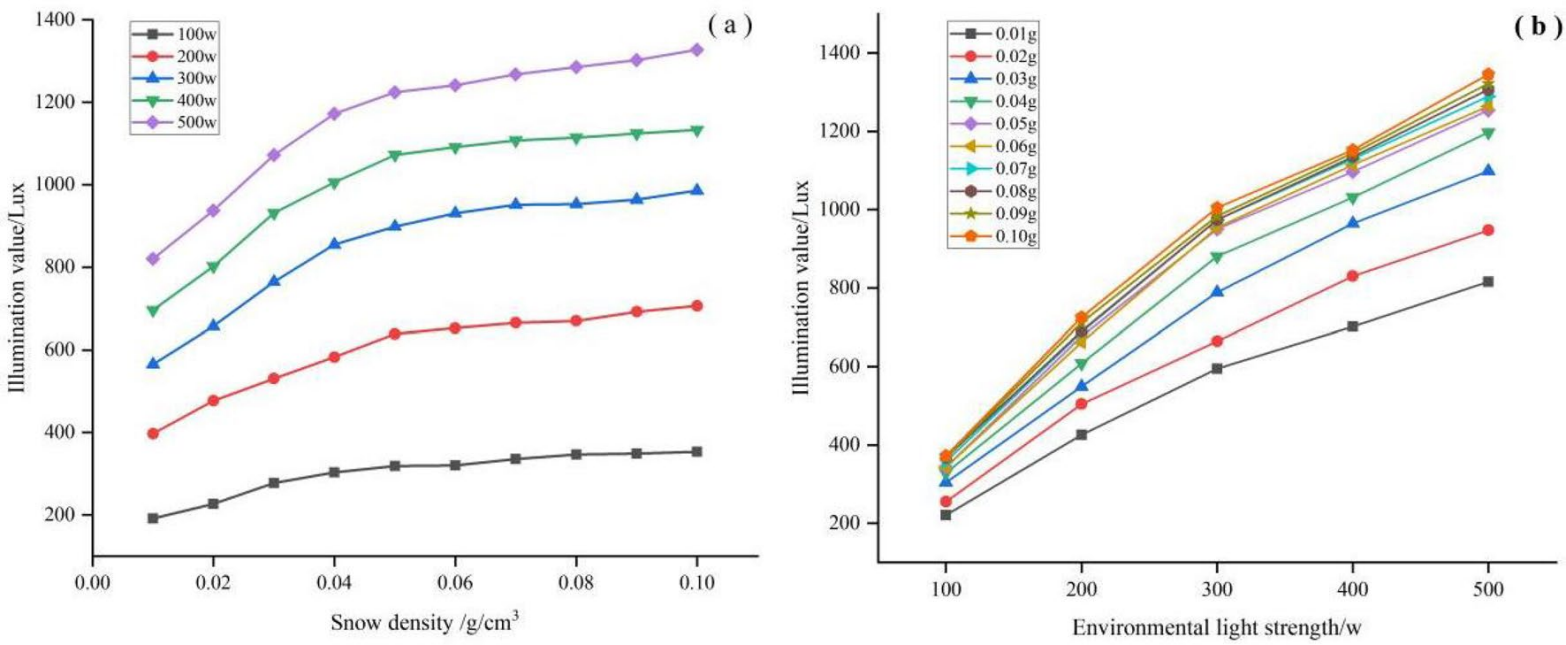
Effects of snow density on the LCE: (**a**) effect of snow density on illumination values under different light conditions; and (**b**) effect of light on illuminance values at different snow densities.

The effect of various snow densities on light intensity under different light levels is shown in Fig. 14a. The results demonstrated that the light levels followed an increasing trend as the snow density increased. The light intensity of various snow densities under different light intensities is shown in Fig. 14b. The results demonstrated that the light values at all levels of snow density also followed an increasing trend as the light intensity increases.

Further analysis shows that as the ambient light intensity increases, the effect on light intensity at different snow densities the rate of change was inconsistent. The lower the snow density, the faster the rate of increase with increasing light intensity. This means that the accumulation of The greater the density of snow, the greater its effect with increasing light intensity (Table 10).

**Table 10:** Equation for the relationship between light intensity and illuminance values at different snow densities.

Snow density/g/cm^3^	Equation	R^2^	Rate of change Lux/w
0.01	Y = 1.557x + 67.142	0.994^a^	1.557
0.02	Y = 1.746x + 96.259	0.991^a^	1.746
0.03	Y = 1.990x + 118.182	0.992^a^	1.990
0.04	Y = 2.161x + 135.480	0.990^a^	2.161
0.05	Y = 2.245x + 157.194	0.987^a^	2.245
0.06	Y = 2.280x + 163.266	0.984^a^	2.280
0.07	Y = 2.305x + 174.045	0.985^a^	2.305
0.08	Y = 2.322x + 177.368	0.987^a^	2.322
0.09	Y = 2.338x + 184.710	0.986^a^	2.338
0.10	Y = 2.375x + 188.588	0.985^a^	2.375

^a^The significance level of the difference in means is 0.01.

#### Effects of snow pollution on the LCE

**Figure 15:**
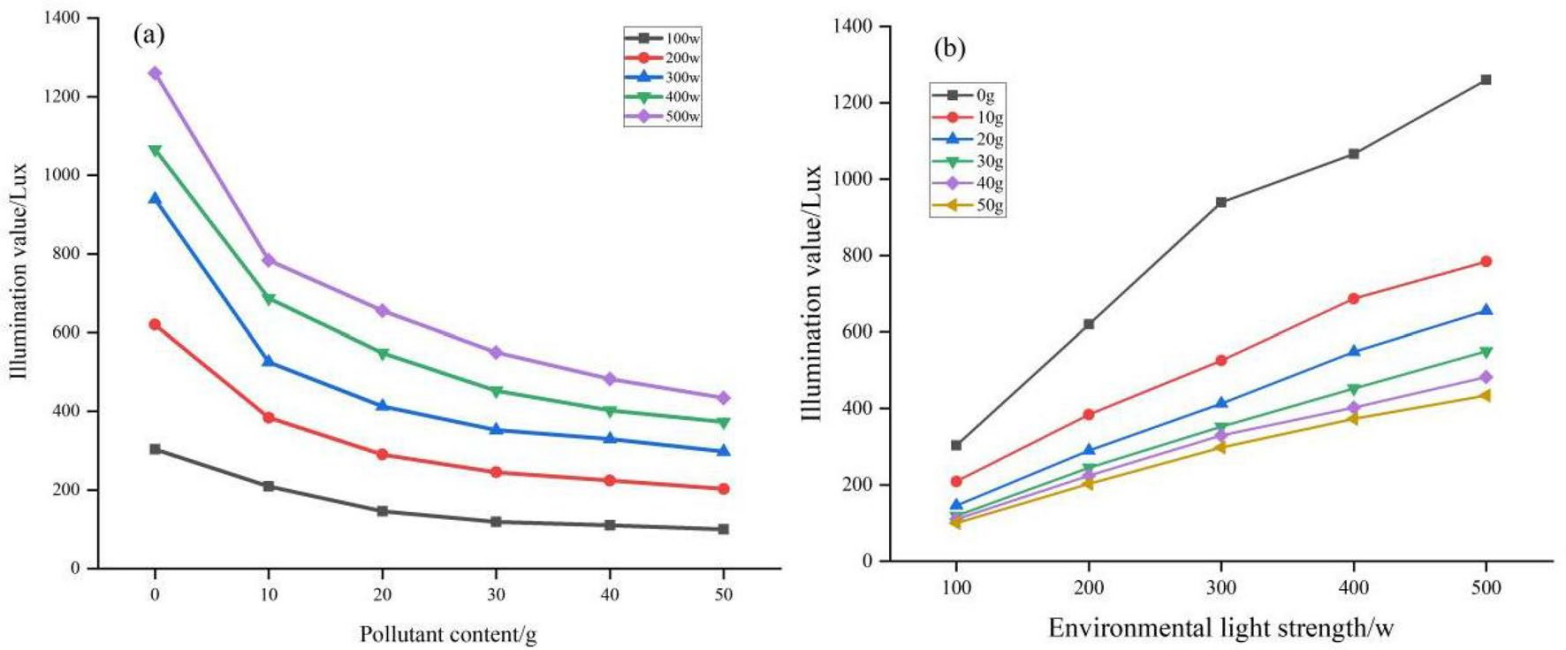
Effects of black carbon in snow on LCE: (**a**) effects of BC pollutants on illuminance values under different light conditions; and (**b**) effects of light on illuminance values under different BC pollutants.

As one of the important factors influencing the impact of snow on the LCE, this study investigated the impact of black carbon (BC), the most active pollutant on the snow surface, on the LCE through controlled experiments. We carried out the experimental design and two-factor interaction test by controlling the BC pollutant content and light level (Fig. 15). We concluded that, under the same light level, the greater the contaminant content of BC was, the weaker the ambient light and the weaker the ambient light intensity (Table 11). At the same BC pollutant content, the light intensity on the snow increased as the light intensity increased. When the snow surface was free of BC contamination, the average light intensity was 882.85 Lux. When BC content was 50 g, the average light intensity was 280.41 Lux, and the ambient light intensity was reduced by 68.24%. When BC content was 10 g, 20 g, 30 g, and 40 g, the ambient light intensity was reduced by 42.98% and 53.12% and 61.58%, 61.58%, and 65.25%. The ANOVA results showed that significant differences in illumination values for different BC content snow at the same light level.

**Table 11:** Equation for the relationship between BC and illuminance values under different light levels.

Illumination/W	Equation	R^2^	Rate of change Lux/w
100	Y = 0.114x^2^−9.553x + 298.604	0.996^a^	0.114
200	Y = 0.243x^2^−19.622x + 595.371	0.985^a^	0.243
300	Y = 0.405x^2^−31.245x + 886.733	0.972^a^	0.405
400	Y = 0.375x^2^−31.348x + 1028.271	0.988^a^	0.375
500	Y = 0.425x^2^−35.933x + 1202.954	0.980^a^	0.425

^a^The significance level of the difference in means is 0.01.

Further analysis shows that the rate of effect of BC concentrations on light intensity shows variability as the ambient light intensity increases (Table 12). It can be seen that the smaller the BC concentration in the snow, the greater the rate of influence on the ambient light level.

**Table 12:** Equation for the relationship between light and illuminance values at different BC concentrations.

BC/g	Equation	R^2^	Rate of change Lux/w
0	Y = 235.803x + 130.197	0.985^a^	235.803
10	Y = 145.193x + 82.447	0.996^a^	145.193
20	Y = 127.670x + 27.757	0.999^a^	127.670
30	Y = 107.037x + 22.757	0.995^a^	107.037
40	Y = 92.343x + 32.950	0.980^a^	92.343
50	Y = 83.807x + 30.253	1.000^a^	83.807

^a^The significance level of the difference in means is 0.01.

## Discussion

This paper provides a preliminary assessment of the effect of snow on photoclimate by means of observational tests and statistical analysis. The study included the effect of snow on daytime and nighttime of LCE. The effects of snow depth, density pollution, etc. on the properties on photoclimate are also included. However, this study is limited to a one-year observation experiment, which simply reveals the linear relationship and effect of snow accumulation on lightness. The mechanism of the effect of snowpack characteristics on light climate needs to be further explored in depth.

1. In the 1960s, Me Zhensheng proposed a formula for comparing the shortwave radiation emitted from various types of ground, and compared the difference in radiation when the ground was covered with snow and when the ground was not covered with snow^48^. He concluded that when the ground was covered with snow, the direct radiation increased by about 20% on a clear day and by more than twice on a cloudy day. These results suggested that at high latitudes, snow had a significant effect on illuminance and thus holds great importance for LCE^49^. Research into the effect of snow on illuminance, however, has been lacking. We conducted a quantitative study of the effect of snow on illuminance using real-world observations.
2. We observed a significant difference between the ground with snow and the ground without snow, with the ground with snow significantly increasing the daytime ambient light level. The daily average ambient illuminance of the ground with snow was 15,424.714 Lux and the daily average ambient illuminance of the ground without snow was 2713.655 Lux, which was 5.684 times higher than the ambient illuminance of the ground without snow, which was an increase of 12,711.059 Lux. The ambient illuminance of the ground with snow was higher than the ambient illuminance of the ground without snow at all times of the day. The effect of snow on illumination is a quadratic parabolic curve with the equation: Y = 393.005 + 2050.578 X−48.922X^2^. The rate of change in snow on illuminance was 48.922 Lux/degree per unit in the solar altitude angle.
3. Snow added to nighttime illuminance in the presence or absence of moonlight. Nighttime illuminance (22:00–4:00 at night) with and without snow when there is no moonlight is 0.010 Lux, 0.005 Lux respectively. Nighttime illuminance with and without snow in the presence of moonlight is 0.213 Lux, 0.010 Lux respectively. With a significant difference in nighttime illuminance with and without moonlight conditions. Snow increased the light level by 0.203 Lux with moonlight and 0.005 Lux without moonlight. From 22:00 to 4:00 at night, at each time point, there was an effect, but that effect was not significantly consistent with time point (P > 0.1).
4. Pollutants emitted by human activities profoundly affect the chemical composition of the snowpack. The direct effect of light-absorbing components (e.g., black carbon, organic carbon, dust, etc.) is to reduce the snow-ice albedo and to warm the snowpack and promote snow-ice melting by absorbing more solar radiation as the snow-ice surface becomes darker. As a result, snow pollution is intensifying under human activities, and its impact on the snow light climate environment should not be underestimated. The light-absorbing substances on the snow surface also include mineral dust, brown carbon, and organic carbon. The quantitative impact of these light-absorbing substances deposited on the snow surface on LCE was not addressed in this study and needs to be explored in further research.

## Conclusion

The conclusions of this study are as follows:

1. Ground with snow significantly improves daytime ambient light levels. The ambient illuminance of the ground with snow is 12,711.059 Lux, 5.684 times higher than the ambient illuminance of the ground without snow.
2. At night, when there is moonlight, the snow increases the illumination of light by 0.203 Lux and in the absence of moonlight, the snow illumination increases by 0.005 Lux.
3. The effect of increasing snow depth on lightness increased in different lightness illumination background, and its rate of increase tends to increase with the increase of snow depth. In different illumination backgrounds, the The rate of increase was 6.534 Lux/cm, 7.769 Lux/cm, 11.697 Lux/cm, 17.726 Lux/cm and 18.070 Lux/cm. As the snow density increases, the effect on light level becomes more significant.
4. For the same background light intensity, different BC contaminants substance content had a significant effect on the light level, with BC pollutant content increases, the effect on the illuminance becomes more significant.

## Data Availability

The datasets generated and/or analysed during the current study are not publicly available due [Project confidentiality] but are available from the corresponding author on reasonable request. Correspondence and requests for data should be addressed to F. Zhang (zhangfan@igsnrr.ac.cn).
